# Community-acquired *Acinetobacter baumannii* bloodstream infection with central nervous system involvement: a case report

**DOI:** 10.3389/fmed.2026.1897823

**Published:** 2026-09-09

**Authors:** Shuai Zhou, Jihong Zhang, Zhixin Ji, Wenzheng He, Qinghai Zhang, Lina Jiang

**Affiliations:** 1 Department of Critical Care Medicine, First Affiliated Hospital of Shandong Second Medical University (Weifang People's Hospital), Weifang, Shandong, China; 2 Department of Neurology, First Affiliated Hospital of Shandong Second Medical University (Weifang People’s Hospital), Weifang, Shandong, China

**Keywords:** *Acinetobacter baumannii*, case, central nervous system infection, community-acquired infection, virus

## Abstract

**Objective:**

This study reports a rare case of community-acquired *Acinetobacter baumannii* bloodstream infection complicated by central nervous system (CNS) involvement, providing insights into its clinical characteristics, diagnosis, and treatment.

**Methods:**

A 76-year-old woman presented with fever, chills, and altered mental status. Blood and cerebrospinal fluid cultures confirmed *A. baumannii* infection, with no evidence of pulmonary involvement.

**Results:**

The patient was successfully treated with a combination of meropenem and both intravenous and intrathecal colistin methanesulfonate.

**Conclusion:**

While *A. baumannii* is primarily associated with hospital-acquired infections, this case highlights its potential to cause severe community-acquired bloodstream infections with CNS involvement.

## Introduction

*Acinetobacter baumannii* is a non-fermentative, oxidase-negative Gram-negative bacillus. It is widely distributed in the natural environment, hospital settings, and human skin and mucosal surfaces, demonstrating strong environmental adaptability and a high risk of multidrug resistance (1). Over the last 30 years, the widespread use of invasive procedures, broad-spectrum antibiotics, immunosuppressants, and ventilators has contributed to the emergence of *A. baumannii* as an important pathogen that is now predominantly associated with nosocomial infections, particularly in immunocompromised or critically ill patients. It is predominantly prevalent in high-risk departments such as the intensive care unit (ICU), neurosurgery, geriatrics, and respiratory medicine and can cause ventilator-associated pneumonia, bloodstream infection, intracranial infection, urinary tract infection, and skin and soft tissue infection, with a persistently high mortality rate (2, 3).

Traditionally, *A. baumannii* infections are predominantly hospital-acquired, and community-acquired infections have long been regarded as rare or extremely uncommon (4). However, epidemiological data over the last decade have shown that the incidence of community-acquired *A. baumannii* infection has increased significantly in tropical, subtropical, and some humid and hot regions (5, 6). Its clinical manifestations are more severe, often characterized by fulminant pneumonia, septic shock, and rapid multiple organ failure. It is easily misdiagnosed as conventional community-acquired pneumonia, leading to initial treatment failure (7, 8). Most reported community-acquired cases are pulmonary infections, and susceptible populations are mostly complicated with diabetes mellitus, chronic obstructive pulmonary disease, alcoholism, renal insufficiency, long-term corticosteroid use, or immunodeficiency (9, 10).

Compared with community-acquired pneumonia, community-acquired *A. baumannii* bloodstream infection is much rarer. Cases of central nervous system (CNS) involvement due to further dissemination originating from a bloodstream infection have been reported only sporadically in domestic and foreign literature (11). Due to the blood–brain barrier, the penetration of antibacterial drugs is low, and lesion clearance is difficult in CNS infections. Once patients develop secondary *Acinetobacter* infection, their underlying disease can rapidly progress to severe meningitis and ventriculitis. This is often accompanied by critical complications including altered mental status, seizures, intracranial hypertension, and cerebral herniation, which lead to extended hospital stays and a substantial rise in disability and mortality rates (12, 13). To date, clinical data on community-acquired *A. baumannii* bloodstream infection with CNS invasion are extremely scarce in China, and there is a lack of typical case summaries and standardized treatment recommendations (14). Owing to insufficient clinical awareness, atypical early clinical manifestations, and the long time required for bacterial culture, such cases are often missed or misdiagnosed, leading to inappropriate empirical anti-infection regimens and delayed treatment (15).

This paper reports one elderly severe case of community-acquired *A. baumannii* bloodstream infection complicated with CNS involvement. The clinical manifestations, laboratory characteristics, imaging changes, treatment strategies, and prognostic outcomes were systematically summarized. Combined with relevant literature, we further discussed its epidemiological features, pathogenic mechanisms, key diagnostic points, and selection of antimicrobial regimens, aiming to improve the ability of clinicians in early recognition and treatment of this disease and provide a reference for clinical diagnosis and management.

## Case

A 76-year-old female patient was admitted directly from her home to the Emergency Department of the First Affiliated Hospital of Shandong Second Medical University (Weifang People's Hospital) due to a persistent high fever lasting 6 h. Upon presentation, she exhibited chills, headache, slurred speech, and progressive confusion, accompanied by one episode of vomiting during the disease course, with no cough or expectoration observed. Neurological examination revealed disturbed consciousness with a Glasgow Coma Scale (GCS) score of 8 points and an impaired ability to follow commands, blunted bilateral pupillary light reflexes, a localized pain response on the left side, and minimal spontaneous movement on the right side, alongside positive meningeal irritation signs (Kernig's and Brudzinski's signs); meanwhile, lung auscultation showed no abnormal findings. The patient presented to the emergency department with an initial body temperature of 38.9 ℃ and was subsequently transferred to the Department of Infectious Diseases after a preliminary clinical assessment. Upon admission to the Department of Infectious Diseases, her vital signs were recorded as a temperature of 39.5 ℃, a heart rate of 116 beats/min, a respiratory rate of 25 breaths/min, a blood pressure of 90/59 mmHg, and a peripheral blood oxygen saturation of 90%–95% under high-flow oxygen inhalation [fraction of inspired oxygen (FiO_2_) 60%, 50 L/min], with an Acute Physiology and Chronic Health Evaluation II (APACHE II) score of 27 points. Arterial blood gas analysis revealed hypoxemia (PaO_2_ 70 mmHg) and hyperglycemia (18.4 mmol/L), and laboratory tests showed markedly elevated inflammatory markers, including a white blood cell count of 22.93 × 10^9^/L (neutrophils 94.4%), a C-reactive protein (CRP) level of 31.7 mg/L, and a procalcitonin (PCT) level of 20.65 ng/mL. Lumbar puncture was performed on the night of April 25 for cerebrospinal fluid (CSF) sampling, and two sets of blood samples were simultaneously collected from separate peripheral venous sites in strict accordance with standard clinical protocols. CSF examination showed dark yellow fluid with an initial pressure of 220 mmH_2_O, a white blood cell count of 637 × 10^6^/L, a protein concentration of 3,681.1 mg/L, and a glucose level of 1.99 mmol/L. Subsequent microbial cultures confirmed the pathogen: CSF culture on April 29 and blood culture on April 30 tested positive for *A. baumannii*. Chest CT examination demonstrated bilateral segmental atelectasis without typical imaging manifestations of pneumonia. The patient had no history of hospitalization or exposure to medical institutions within the previous 6 months and had not received broad-spectrum antibiotic treatment prior to admission. She had a 13-year history of poorly controlled hypertension, with a maximum recorded blood pressure of 220/100 mmHg, and had developed hemiplegia secondary to cerebral hemorrhage 2 years prior to this admission.

**Figure 1:**
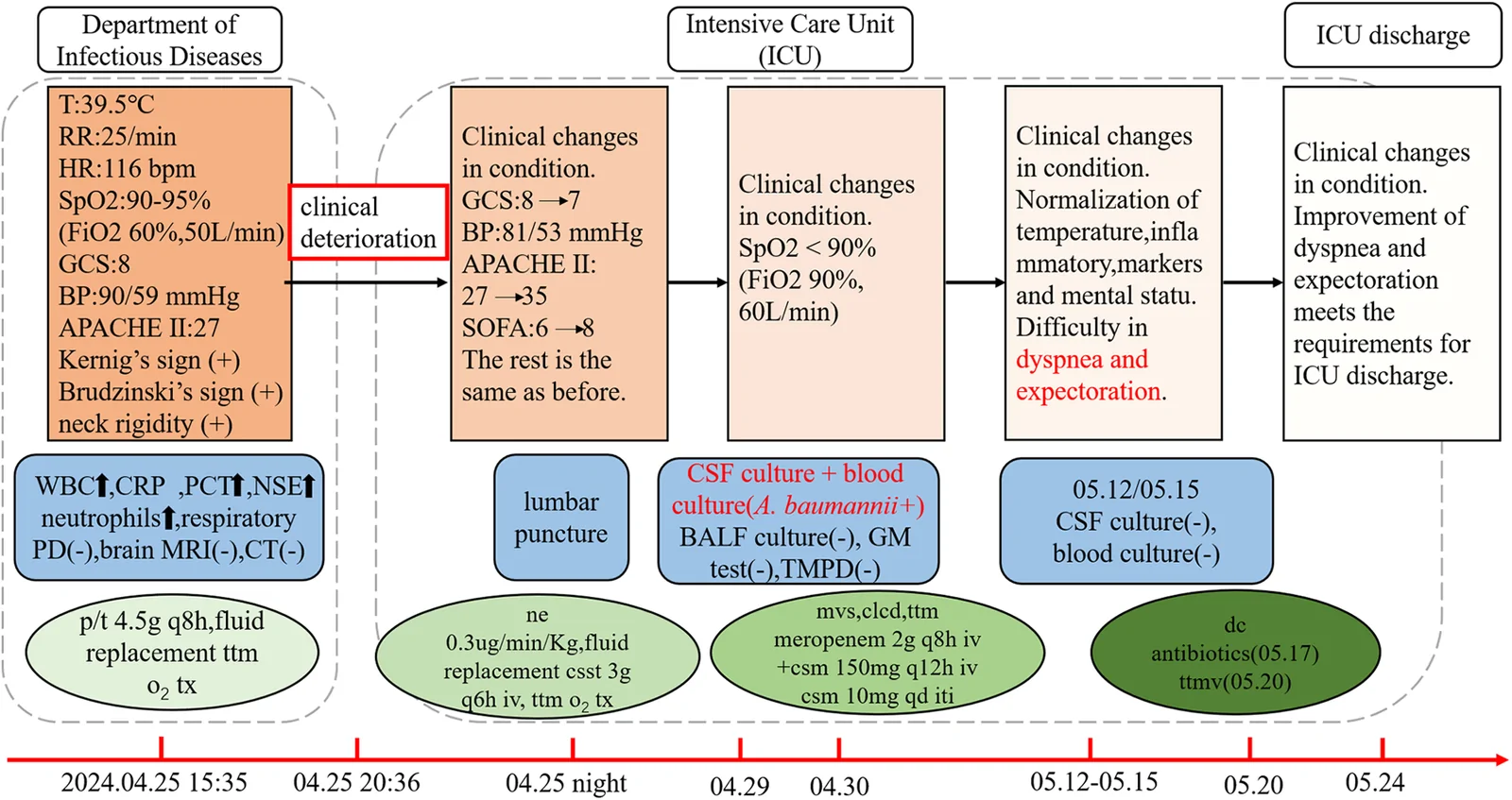
Clinical timeline and management course of a patient with severe *A. baumannii* meningitis progressing to critical illness. T, temperature; RR, respiratory rate; HR, heart rate; bpm, beats per minute; SpO_2_, pulse oxygen saturation; FiO_2_, fraction of inspired oxygen; GCS, Glasgow Coma Scale; APACHE II, Acute Physiology and Chronic Health Evaluation II; SOFA, Sequential Organ Failure Assessment; BP, blood pressure. WBC, white blood cell; CRP, C-reactive protein; PCT, procalcitonin; NSE, neuron-specific enolase; PD, pulmonary disease; MRI, magnetic resonance imaging; CT, computed tomography; CSF, cerebrospinal fluid; BALF, bronchoalveolar lavage fluid; GM test, galactomannan assay; TMPD, *Tuberculous mycobacteria* detection. p/t, piperacillin–tazobactam; q8h, every 8 h; ttm, targeted temperature management; O_2_ tx, oxygen therapy; ne, norepinephrine; csst, cefoperazone–sulbactam; mvs, mechanical ventilation support; clcd, closed-loop sedation delivery; csm, colistin; iv, intravenous administration; iti, intraventricular injection; dc, discontinue.

The patient initially received empirical antibacterial therapy with piperacillin–tazobactam (4.5 g intravenous infusion every 8 h) combined with comprehensive supportive treatment. This broad-spectrum regimen was initiated for suspected severe systemic bacterial sepsis, as definite CNS infection and pathogen identification were not confirmed at admission, and empirical anti-infective coverage was prioritized to control life-threatening systemic inflammation. However, the clinical condition of the patient gradually deteriorated, characterized by persistent high fever, aggravated consciousness disturbance (GCS score decreased from 8 to 7 points), and progressive hemodynamic instability, leading to urgent transfer to the ICU. The empirical anti-infective regimen was then upgraded to cefoperazone–sulbactam (3 g intravenously every 6 h). Considering the persistent hypoxemia and progressive neurological deterioration of the patient, endotracheal intubation combined with mechanical ventilation and lumbar catheter drainage were performed immediately. After *A. baumannii*-associated CNS infection was definitively confirmed, the treatment regimen was further adjusted to meropenem (2 g intravenously every 8 h) combined with colistin methanesulfonate (150 mg intravenously every 12 h plus 10 mg intrathecally once daily) based on the antimicrobial susceptibility test results (Table 1). Following the standardized anti-infective and supportive treatment, the patient's clinical symptoms improved gradually, body temperature returned to normal, and inflammatory markers decreased significantly. Repeat CSF and blood cultures performed on 12 and 15 May were negative. Antibiotic treatment was discontinued on 17 May, and the lumbar cistern drainage catheter was removed on the following day. Due to persistent respiratory insufficiency, tracheotomy was performed on 20 May. With the gradual recovery of respiratory function, the patient was transferred to the rehabilitation department for further treatment on 24 May. At the 1-month follow-up in June 2024, the patient regained full consciousness but remained dependent on tracheotomy ventilation and nasogastric feeding, with an Activity of Daily Living (ADL) score of 55 points, indicating moderate functional impairment. At the 6-month follow-up in November 2024, the patient achieved further functional recovery, with an ADL score of 75 points; the tracheotomy tube was retained, and the patient tolerated a semi-liquid oral diet. The detailed disease progression and full treatment course of the patient are illustrated in Figure 1.

**Table 1:** Antimicrobial susceptibility testing results of the *A. baumannii* isolate.

Antimicrobial agents	MIC (µg/mL)	Zone diameter (mm)	Result interpretation
Piperacillin–tazobactam	≥128	—	R
Ceftazidime	≥64	—	R
Cefepime	≥64	—	R
Cefoperazone–sulbactam	—	12	R
Imipenem	≥16	—	R
Meropenem	≥16	—	R
Ciprofloxacin	≥4	—	R
Levofloxacin	≥8	—	R
Amikacin	≥64	—	R
Tobramycin	≥16	—	R
Doxycycline	≥16	—	R
Minocycline	—	14	I
Trimethoprim–sulfamethoxazole	≥320	—	R
Polymyxin B	—	18	S

R, resistant; I, intermediate; S, susceptible; MIC, minimum inhibitory concentration

## Discussion

*A. baumannii* infections are predominantly hospital-acquired, while community-acquired cases are rare and mostly originate from the lungs (7, 16). The present case involved a rare community-acquired *A. baumannii* bloodstream infection with CNS involvement, confirmed by positive blood and CSF cultures. Chest CT revealed no pulmonary consolidation or infiltrates, excluding pneumonia as the primary infectious focus. Comprehensive physical examination, including careful inspection of the skin and soft tissues, otoscopic examination, and sinus percussion, revealed no signs of localized infection such as cellulitis, abscess, sinusitis, or otitis media. In the absence of identifiable local infectious foci, hematogenous dissemination of cryptogenic *A. baumannii* bacteremia is considered the most plausible mechanism for secondary intracranial infection, which represents an uncommon route of transmission for community-acquired *A. baumannii* meningitis.

Although the calculated PaO_2_/FiO_2_ ratio under the current oxygen support was approximately 117, which falls within the severe Acute Respiratory Distress Syndrome (ARDS) category, the diagnosis of ARDS according to the Berlin definition requires the presence of bilateral opacities consistent with pulmonary edema not fully explained by effusion, atelectasis, or nodules. In our patient, chest CT revealed only bilateral segmental atelectasis without consolidation, ground-glass opacities, or pleural effusion, thereby definitively excluding the radiographic criteria for ARDS. Therefore, the hypoxemia in this case was primarily attributed to hypoventilation secondary to impaired consciousness and to ventilation–perfusion mismatch caused by atelectasis, rather than diffuse alveolar damage. Several lines of evidence support this mechanistic interpretation. First, the patient presented with a GCS score of 8 on admission, indicating substantial impairment of consciousness; this likely led to reduced central respiratory drive, inadequate tidal volume, and subsequent hypoventilation. Second, she had a history of long-term immobility due to hemiplegia from a previous cerebral hemorrhage, predisposing her to dependent segmental atelectasis, which in turn caused ventilation–perfusion mismatch and contributed to hypoxemia. Third, the clinical course further corroborates this explanation: after endotracheal intubation with mechanical ventilation and active pulmonary physiotherapy, her oxygenation progressively improved in parallel with the recovery of her conscious state. This pattern of improvement would not be expected if diffuse alveolar damage (i.e., ARDS) had been the primary underlying pathology, as ARDS typically requires a more protracted recovery period and is often associated with persistent radiographic abnormalities.

In tropical and subtropical regions, several cases have been documented. Brazil reported a rare case of community-acquired pneumonia caused by this pathogen, highlighting diagnostic challenges due to its multidrug-resistant nature and rapid progression (17). Korea reported an outbreak of community-acquired *A. baumannii* pneumonia with a high fatality rate, which was more severe than its hospital-acquired counterpart (18). A systematic study conducted in northeastern Thailand described the clinical features of community-acquired *A. baumannii* pneumonia, highlighting its unique manifestations as an emerging lethal disease (19). In Western countries, although such infections remain uncommon, sporadic cases have been reported in the United States and France. As demonstrated by case reports from the United States (20) and the relevant studies from France (21), although the incidence rate is lower in the temperate regions of Europe and America, severe community-acquired *A. baumannii* infections can still occur in immunocompromised patients and long-term heavy drinkers. Collectively, these reports, together with our case, underscore that community-acquired *Acinetobacter* infection, although rare, is a global phenomenon that warrants clinical awareness beyond traditionally recognized endemic areas.

Once *A. baumannii* causes meningitis or ventriculitis, it frequently leads to increased intracranial pressure, disturbance of consciousness, and refractory infection, with a marked increase in mortality (12, 22). On admission, the patient presented with high fever, coma, and positive meningeal irritation signs. CSF examinations showed typical purulent changes, including a markedly elevated white blood cell count, an extremely high protein level, and a significantly decreased glucose level, which were consistent with the diagnostic criteria for severe CNS infection. The core therapeutic challenge of CNS infection lies in the blood–brain barrier, which limits the penetration of antibacterial agents, making it difficult for conventional intravenous administration to achieve effective bactericidal concentrations in CSF (23). Therefore, drugs with favorable blood–brain barrier permeability should be preferred (12, 24, 25). For patients with severe infection, intraventricular or intrathecal administration can be considered (26, 27). Colistin achieves low CSF concentrations when administered intravenously, whereas intrathecal injection enables direct delivery to the site of infectious and exerts rapid bactericidal effects (9). Studies have confirmed that meropenem can partially penetrate the blood–brain barrier under inflammatory conditions and exerts a synergistic effect when combined with colistin (10). In this case, a regimen of intravenous meropenem plus intravenous and intrathecal colistin methanesulfonate was used, combined with continuous lumbar cistern drainage. The infection was effectively controlled. After 17 days of treatment, the patient gradually improved and eventually recovered.

Risk factors for community-acquired *A. baumannii* infection include advanced age, diabetes mellitus, chronic pulmonary disease, immunodeficiency, and a history of cerebrovascular disease (28, 29). Distinct risk factors are identified among community-acquired, healthcare-associated, and nosocomial *A. baumannii* infections. Heavy alcohol consumption and smoking are well-established predisposing factors for community-acquired infection, as documented in previous clinical studies (4, 30). In contrast, major risk factors for hospital-onset infection include endotracheal intubation, prior broad-spectrum antibiotic administration, prolonged hospital stay, and central venous catheter placement (31), which are closely linked to invasive medical interventions and nosocomial transmission pathways. The patient in this case had hypertension, sequelae of previous cerebral hemorrhage, and long-term limb dysfunction, placing her in a high-risk population with weakened immune function and susceptibility to community-acquired infection. *A. baumannii* is a classic opportunistic pathogen, predominantly causing infections in immunocompromised individuals (32). However, accumulating evidence indicates that it can also cause severe community-acquired infections in patients with certain predisposing conditions, even in the absence of classic immunosuppression (33). Our case illustrates that under predisposing conditions such as cerebrovascular sequelae, *A. baumannii* can also cause severe community-acquired CNS infection (34, 35). The patient had been hospitalized for cerebral hemorrhage 2 years earlier, and previous *A. baumannii* colonization could not be completely excluded. Although *A. baumannii* is highly resilient under harsh conditions and can survive on dry surfaces for approximately 100 days, as previously reported (36), there have been no reports of survival exceeding 2 years. Therefore, the infection in this case was determined to be acquired from the natural community environment. This finding clinically suggests that even without a recent hospitalization history, elderly patients with underlying cerebrovascular diseases may still develop community-acquired *A. baumannii* bloodstream infection complicated with CNS involvement, for which close clinical vigilance should be maintained.

Due to the rarity of community-acquired cases, atypical early symptoms, and the prolonged time required for bacterial culture, such infections are easily misdiagnosed clinically as common CNS infections caused by *Streptococcus pneumoniae*, *Neisseria meningitidis*, and other pathogens, leading to deviations in initial anti-infective regimens (37). This bacterium exhibits strong antibiotic resistance, and diagnosis mainly relies on bacterial culture and metagenomic next-generation sequencing (mNGS). Therefore, in future diagnosis and treatment, bacteriological examinations should be initiated as early as possible to confirm the diagnosis, clinical awareness of *A. baumannii* should be enhanced, and early detection, early diagnosis, and early treatment should be achieved. This will help prevent the overuse of antibiotics and significantly improve the quality of life of patients.

We admit that, apart from chest CT, no other examinations, such as sinus puncture, abdominal imaging, or echocardiography, were conducted to completely rule out other potential hidden primary lesions—this is a limitation of this case report. In addition, blood lactate level was not measured upon admission, which precluded a complete quantitative assessment of septic shock severity according to the Sepsis-3 criteria and limited our objective evaluation of tissue hypoperfusion at initial presentation. The limitations of this study also include its being a single-center case report with a small sample size, which prevents the determination of the optimal treatment plan, dosage, and prognostic factors for such infections. In the future, multicenter retrospective or prospective studies are needed to further explore the epidemiology, risk factors, and optimal antimicrobial regimens for community-acquired *A. baumannii* bloodstream infection complicated by CNS involvement.

## Conclusion

Community-acquired *A. baumannii* can cause severe CNS infection with bloodstream infection as the primary focus, which is characterized by dangerous clinical manifestations and a high misdiagnosis rate. Elderly patients with underlying cerebrovascular diseases are high-risk groups. Early etiological confirmation, combined intravenous and intrathecal antimicrobial therapy, and continuous CSF drainage can significantly improve the prognosis. Clinicians should enhance their awareness of this disease to achieve early identification, early diagnosis, and precise treatment.

## Data Availability

The original contributions presented in the study are included in the article/Supplementary Material; further inquiries can be directed to the corresponding author.
